# Curcumin-Encapsulated Polymeric Micelles Suppress the Development of Colon Cancer *In Vitro* and *In Vivo*

**DOI:** 10.1038/srep10322

**Published:** 2015-05-18

**Authors:** Xi Yang, Zhaojun Li, Ning Wang, Ling Li, Linjiang Song, Tao He, Lu Sun, Zhihan Wang, Qinjie Wu, Na Luo, Cheng Yi, Changyang Gong

**Affiliations:** 1 Department of Medical Oncology, Cancer Center, State Key Laboratory of Biotherapy/Collaborative Innovation Center of Biotherapy, West China Hospital, Sichuan University, Chengdu, 610041, P. R. China; 2 School of Medicine, Nankai University, Tianjin, 300071, China

## Abstract

To develop injectable formulation and improve the stability of curcumin (Cur), Cur was encapsulated into monomethyl poly (ethylene glycol)-poly (ε-caprolactone)-poly (trimethylene carbonate) (MPEG-P(CL-*co*-TMC)) micelles through a single-step solid dispersion method. The obtained Cur micelles had a small particle size of 27.6 ± 0.7 nm with polydisperse index (PDI) of 0.11 ± 0.05, drug loading of 14.07 ± 0.94%, and encapsulation efficiency of 96.08 ± 3.23%. Both free Cur and Cur micelles efficiently suppressed growth of CT26 colon carcinoma cells *in vitro*. The results of *in vitro* anticancer studies confirmed that apoptosis induction and cellular uptake on CT26 cells had completely increased in Cur micelles compared with free Cur. Besides, Cur micelles were more effective in suppressing the tumor growth of subcutaneous CT26 colon *in vivo*, and the mechanisms included the inhibition of tumor proliferation and angiogenesis and increased apoptosis of tumor cells. Furthermore, few side effects were found in Cur micelles. Overall, our findings suggested that Cur micelles could be a stabilized aqueous formulation for intravenous application with improved antitumor activity, which may be a potential treatment strategy for colon cancer in the future.

Cancer is one of the most severe diseases with increasing morbidity and mortality every year around the world. According to statistics in the United States, colorectal cancer is the world most common cancer and the third leading cause of cancer death in both male and female^1^,^2^. In spite of the development of novel powerful treatment, chemotherapy still plays an important role in the management of colon carcinoma. However, drug toxicity and undesirable side effects are the major impediments to a successful chemotherapeutic regimen, such as nausea and vomiting, diarrhea, white blood cell decreased, anemia, fatigue, nerve damage, pain, and skin reactions etc.^3^,^4^.

Curcumin (Cur), known as diferuloylmethane or 1,7-bis (4-hydroxy-3-methoxyphenyl) -1,6-hepatadiene-3,5-dione, is a nature yellow colored and low molecular weight polyphenol compound purified from the rhizome of the plant *Curcuma longa*^5^. Cur has been found as a component of many traditional medicines for a long time in Southeast Asia countries (India and China). Cur has various pharmacological activities, such as anti-inflammatory, anti-oxidant, and anti-tumor effects etc.^6^. Particularly, Cur has been demonstrated efficacy as an anticancer agent for many types of malignancies, including colorectal, breast, lung, prostate, and pancreatic carcinoma^7^. In addition, Cur has been under investigation in human clinical trials for many years and has indicated clinical benefits for patients with colorectal cancer and pancreatic cancer^8^,^9^,^10^,^11^,^12^,^13^. More importantly, to date, neither animals nor human studies have found any toxicity or side effects of Cur^14^,^15^,^16^. Therefore, the natural herbal Cur is less toxic than other chemotherapeutic agents in the treatment of cancer. Despite of the safety and the broad pharmacological effects of Cur, the application in clinic has been hampered due to its extremely low aqueous solubility, instability, poor oral bioavailability and rapid metabolism^17^,^18^.

Some attempts have been made to achieve increased Cur solubilization and protect Cur against inactivation by hydrolysis. So far the nanotechnology is considered as one of the most important methods to design and develop nano-sized delivery systems for Cur including liposomes, conjugates, solid dispersions, peptide carriers, polymeric nanoparticles and micelles^13^. In recent years, micelles have attracted increasing attentions in drug delivery and cancer treatment as nanocarriers. Polymeric micelles can improve aqueous formulations of hydrophobic drugs, prolong their circulation time *in vivo*, enhance the cellular uptake, and passively target at tumor regions by the enhanced permeability and retention (EPR) effect^19^,^20^. Thus, we have recently developed a polymeric micelles formulation of Cur (Cur micelles) which can efficiently enhance the systemic bioavailability.

Previously, Cur-loaded monomethyl poly (ethylene glycol)-poly (ε-caprolactone) (MPEG-PCL) micelles were developed in laboratory. However, X-ray diffraction spectra confirmed that the stronger crystallization of poly (ε-caprolactone) (PCL) was responsible for the micelles instability^21^. We hypothesized that non-crystallization trimethylene carbonate (TMC) in copolymer might inhibit the crystallization of PCL to improve the micelles stability. In order to enhance the water solubility and make it avaliable for injection, Cur loaded into a promising monomethyl poly (ethylene glycol)-poly (ε-caprolactone)-poly (trimethylene carbonate) (MPEG-P(CL-*co*-TMC)) micelles. Then, the cytotoxicity of Cur micelles *in vitro* and the anti-tumor activity in a mouse model of colon cancer were investigated in our article.

## Results

### Preparation and Characterization of Cur Micelles

The biodegradable MPEG-P(CL-*co*-TMC) copolymer was successfully synthesized by ring-opening polymerization of ε-CL and TMC initiated by MPEG using stannous octoate as catalyst. The number-average molecular weights (*M*_n_) of MPEG-P(CL-*co*-TMC) copolymer caculated by ^1^*H*-NMR was 4292 (2000-1836-456).

Cur micelles were peraped by a one-step solid dispersion method as illustrated in Supplementary Fig. 1. During this process, Cur and MPEG-P(CL-*co*-TMC) copolymer co-evaporated as amorphous substance, and co-dissoved in narmal saline (NS) to form core-shell structured Cur micelles. In this work, 15/85 of the Cur/MPEG-P(CL-*co*-TMC) copolymer weight ratio in feed was chosen for future application and characterized in detail.

**Figure 1:**
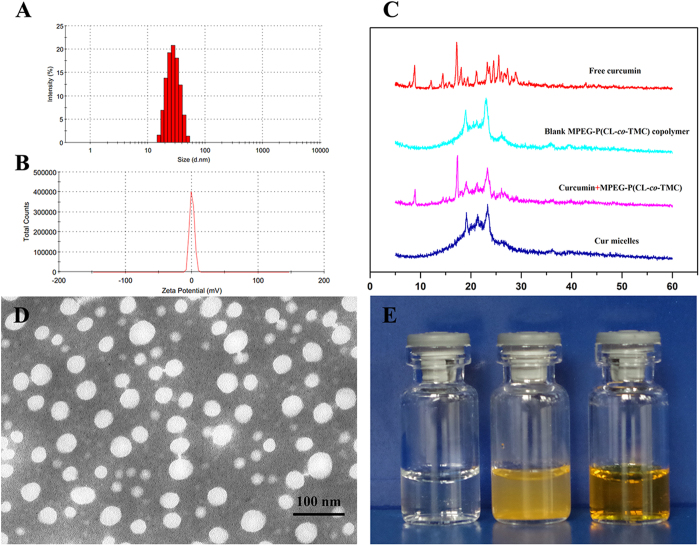
Characterization of Cur micelles. (**A**) Paricle size distribution of Cur micelles; (**B**) Zeta potential of Cur micelles; (**C**) XRD analysis of free Cur, blank MPEG-P(CL-*co*-TMC) copolymer, Cur + MPEG-P(CL-*co*-TMC) copolymer, Cur micelles; (**D**) TEM image of Cur micelles; (**E**) Appearance of blank MPEG-P(CL-*co*-TMC) micelles (left), free Cur in water (middle), and Cur micelles (right).

The drug loading (DL) and encapsulation efficiency (EE) were 14.07 ± 0.94% and 96.08 ± 3.23%, respectively. Figure 1A and B showed that the average particle size of Cur micelles was about 27.6 ± 0.7 nm with a poly dispersity index (PDI) of 0.11 ± 0.05 and a zeta potential of 0.11 ± 0.34 mV.

When the in-feed ratio of Cur/MPEG-P(CL-*co*-TMC) copolymer was 15/85, the prepared Cur micelles kept at 4 or 37 °C were relatively stable within 48 h or 6 h by the observation of aggregates, respectively. The results of the particle size distribution changes at 4 °C or 37 °C showed that the particle size of Cur-encapsulated micelles was relatively stable in 48 h (Supplementary Fig. 2A). The Supplementary Fig. 2B and C exhibited that the EE and DL of Cur decreased slowly within 48 h or 6 h at 4 °C or 37 °C indicating the relatively stable of Cur micelles solution. However, the DL and EE of Cur after 6 h decreased rapidly at 37 °C because of the large precipitation of Cur from Cur micelles.

To investigate the crystallinity of micelles-encapsulated Cur, X-ray diffraction (XRD) analyses were performed on the Cur micelles. The XRD patterns were given in Fig. 1C, including free Cur, blank MPEG-P(CL-*co*-TMC) copolymer, Cur + MPEG-P(CL-*co*-TMC) copolymer, Cur micelles. In XRD spectra of free Cur and Cur + MPEG-P(CL-*co*-TMC), a number of peaks were observed (in the 2*θ* range of 10–30°) implying its crystalline nature, while there were no such Cur crystalline peaks in Cur micelles. In addition, the characteristic crystalline peaks of the PCL (2*θ = *21.5 and 23.5) decreased in blank MPEG-P(CL-*co*-TMC) copolymer compared with blank MPEG-PCL copolymer (the data not showed)^21^,^22^. Herein, the results suggested that Cur could form amorphous complex or disorded-crystalline phase in the Cur micelles, and TMC in copolymer could inhibit the crystallization of PCL.

Transmission electron microscope (TEM, H-6009IV, Hitachi, Japan) image of the Cur micelles was shown in Fig. 1D, which demonstrated a distinct spherical outline and its well-dispersed in aqueous solution. The appearance of blank MPEG-P(CL-*co*-TMC) micelles (left), free Cur in water (middle), and Cur micelles (right) were presented in Fig. 1E. The clear and transparent solution of blank micelles and Cur micelles could be observed implying its well-dispersed in aqueous solution. In contrast, free Cur formed a turbid yellow suspension in water indicating its poorly-dissolved in water. Consequently, loaded Cur into MPEG-P(CL-*co*-TMC) micelles could result in a homogenous and stable dosage form in aqueous media making Cur intravenously injectable *in vivo*.

### *In Vitro* Release Study

**Figure 2:**
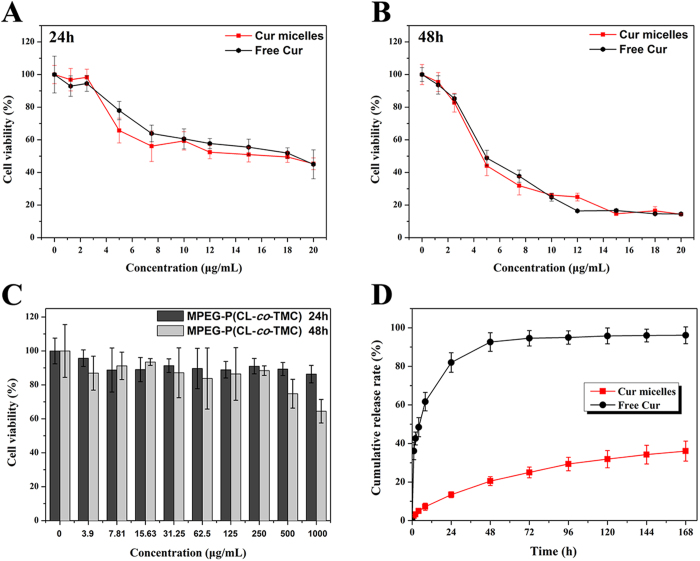
Cytotoxicity studies of free Cur and Cur micelles on CT26 cells for 24 h (**A**) or 48 h (**B**). (**C**) Cytotoxicity evaluation of blank MPEG-P(CL-*co*-TMC) copolymer on CT26 cells for 24 h and 48 h. (**D**) *In vitro* release behavior of Cur from free Cur or Cur micelles, respectively.

The *in vitro* release profiles of free Cur and Cur micelles were studied by a dialysis method. Cur released from free Cur or Cur micelles was continuously monitored in 7 days. As illustrated in Fig. 2D, free Cur showed a very fast release behavior compared with the much slower cumulative release rate of Cur micelles. Only 20% of the loaded Cur was slowly released from micelles in the first 48 h, while approximately 92% of Cur was rapidly released into the outside PBS in free Cur group during the same period. The cumulative release rate of Cur micelles was 36.05 ± 5.21% over the period of 7 days, which was much lower than free Cur (96.12 ± 4.34, *p* < 0.05). Thus, this controlled release of Cur from Cur micelles revealed that their applicability as a drug delivery system could minimize the exposure of healthy tissues and enhance the accumulation of anti-cancer drugs in tumor regions.

### Cytotoxicity Study

To examine the cytotoxicity of free Cur, Cur micelles, and blank MPEG-P(CL-*co*-TMC) micelles, we performed on CT26 colon carcinoma cells *in vitro*. As shown in Fig. 2 A and B cell viability was measured by the MTT assay at 24 h and 48 h post treatment by free Cur and Cur micelles indicating significant concentration dependence. Half-maximal inhibitory concentration (IC_50_) of free Cur and Cur micelles for 24 h were 16.67 μg/mL and 14.22 μg/mL, respectively. Moreover, the IC_50_ of free Cur for 48 h was 5.63 μg/mL, and Cur micelles was 5.50 μg/mL. Although encapsulated Cur in MPEG-P(CL-*co*-TMC) micelles could slightly enhance the cytotoxic activity of Cur compared with free Cur, no significant differences were observed between Cur micelles and free Cur. The results indicated that both free Cur and Cur micelles efficiently suppressed growth of CT26 colon carcinoma cells *in vitro*.

In addition, as presented in Fig. 2C, the viabilities of CT26 cells cultured with blank MPEG-P(CL-*co*-TMC) micelles were higher than 86.34% or 64.46% for 24 h or 48 h at a high concentration of 1000 μg/mL. Therefore, the blank MPEG-P(CL-*co*-TMC) micelles had little cytotoxic as a drug delivery carrier.

### Apoptosis Induction of Cur Micelles *In Vitro*

**Figure 3:**
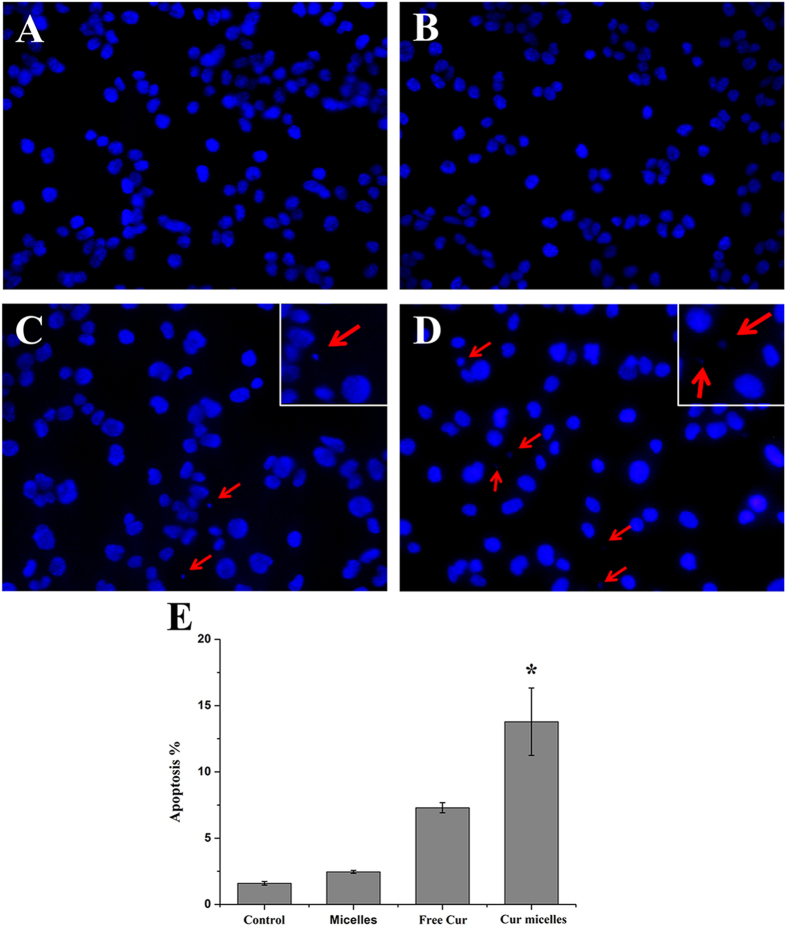
Fluorescent microscopy of apoptotic cells induced by control group (**A**), blank micelles (**B**), free Cur (**C**), Cur micelles (**D**). Nuclei were stained blue with DAPI, and arrows point to the apoptotic body. Induction of apoptosis by free Cur and Cur micelles on CT26 cells (**E**).

DAPI staining of DNA is a morphological method of detecting apoptosis. Especially, apoptotic bodies can be easily observed by fluorescence microscopy, small sealed membrane vesicles, which produced from cells undergoing apoptosis. As shown in Fig. 3A–D, within the same dose, the image of cells treated with Cur micelles presented a relatively increase in apoptotic bodies compared with free Cur, blank MPEG-P(CL-*co*-TMC) micelles (blank micelles) or control group. In addition, flow cytometric (FCM) assay was performed to quantitatively examine apopotosis induction of Cur micelles in CT26 cells by observing sub-G1 (apopototic) cells. The results demonstrated that Cur micelles treated cell populations were 13.79% apopotic, 7.30% in free Cur (*p* < 0.05), 2.46% in blank micelles (*p* < 0.05) and 1.59% in control group (*p* < 0.05), respectively (Fig. 3E). Here, Cur micelles induced a large amount of apopototic cells than other groups *in vitro*.

### Cellular Uptake of Cur Micelles *In Vitro*

**Figure 4:**
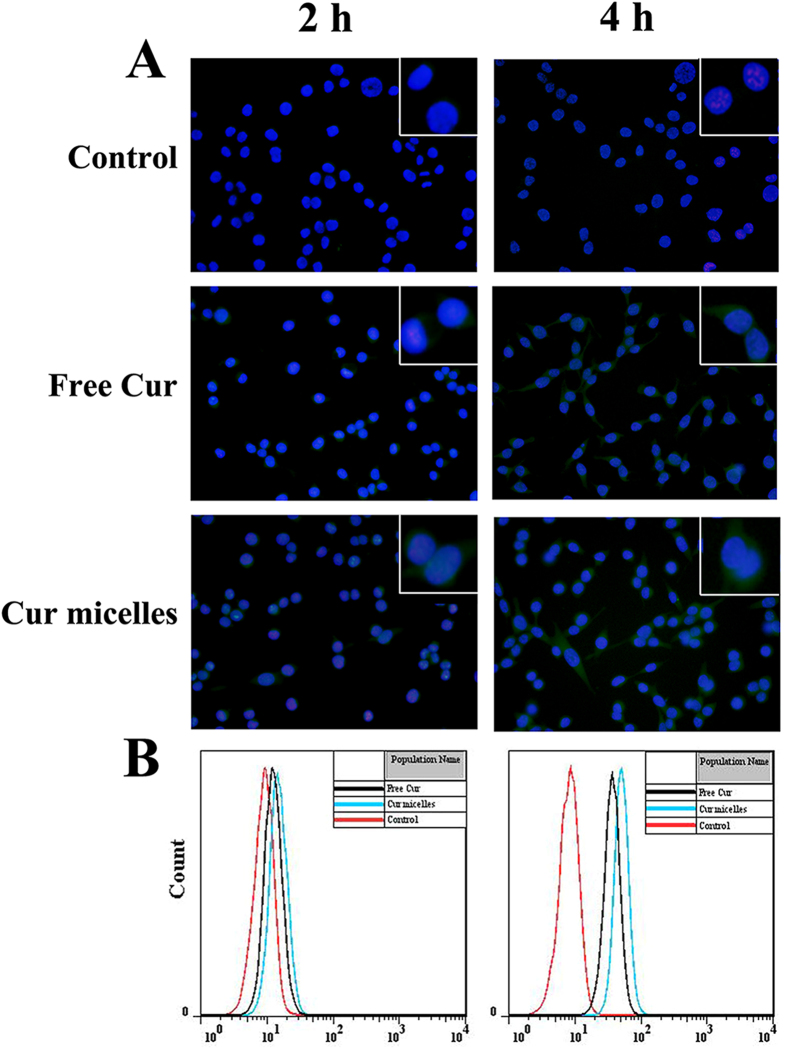
Cellular uptake of Cur micelles. (**A**) Fluorescent images of cells treated with control, free Cur, Cur micelles for 2 h and 4 h, respectively. Nuclei were stained blue with DAPI, and cellular distribution of Cur was shown as green fluorescence in the cytosol. (**B**) Flow cytometeric histograms by free Cur and Cur micelles for 2 h and 4 h on CT26 cells, respectively.

To explore the mechanisms of exerted cytotoxicity and apoptosis effect of Cur micelles, we used the cellular uptake of free Cur and Cur micelles on CT26 cells by fluorescence microscopy and FCM. Figure 4A showed the images of cells treated with control, free Cur and Cur micelles for 2 h and 4 h, respectively. Medium without treatment drug regarded as control group did not present any fluorescence at any time. In free Cur, a very slight fluorescence was observed at 2 h after incubation and green fluorescence was increased after 4 h incubation. By contrast, Cur micelles could rapidly accumulate in the cytosol over 2 h incubation which was revealed by the bright green fluorescence. A more bright green fluorescence in cytosol was investigated after 4 h. Furthermore, the increased cellular uptake of Cur micelles was also determined by FCM. As illustrated in Fig. 4B, the fluorescence intensity of CT26 cells treated with Cur micelles after 2 h and 4 h incubation was much stronger than free Cur. In the Supplementary Fig. 2D, either the mean fluorescence intensity at 2 h or 4 h in Cur micelles (19.22 ± 1.05 and 52.17 ± 0.97) was much higher than those in the free Cur (13.19 ± 0.15 and 40.78 ± 0.26, *p* < 0.05) and control (9.16 ± 0.07 and 8.88 ± 0.04, *p* < 0.05), respectively.

### *In Vivo* Antitumor Activity

**Figure 5:**
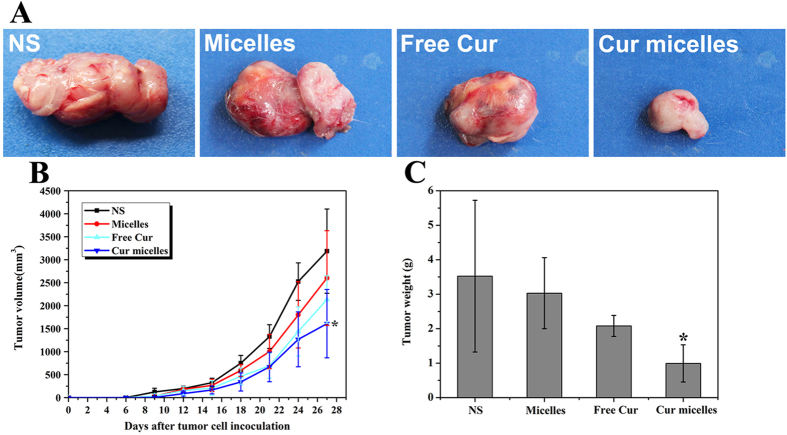
Cur micelles inhibited growth in subcutaneous CT26 model. (**A**) Representative photographs of subcutaneous tumors in each group; (**B**) The tumor growth curves of each group in tumor-bearing mice; (**C**) Weight of subcutaneous in each group.

In order to compare *in vivo* antitumor effect of Cur micelles with free Cur, blank micelles and NS, subcutaneous CT26 model was performed in this study. As shown in Fig. 5A, we have chosen the most representative images of tumors based on the volume and weight in each group. In Fig. 5B, tumor volume of day 27 in Cur micelles group was significant lower than free Cur (*p* < 0.05), blank micelles (*p* < 0.05), NS group (*p* < 0.05), respectively. In addition, Fig. 5C showed the weight of tumor in each group. Tumor weight in Cur micelles group (0.99 ± 0.61 g) was significant lower than free Cur (2.08 ± 0.69 g, *p* < 0.05), blank micelles (3.03 ± 1.16 g, *p* < 0.05), NS group (3.52 ± 1.93 g, *p* < 0.05), respectively.

### Determination of Tumor Cell Proliferation

**Figure 6:**
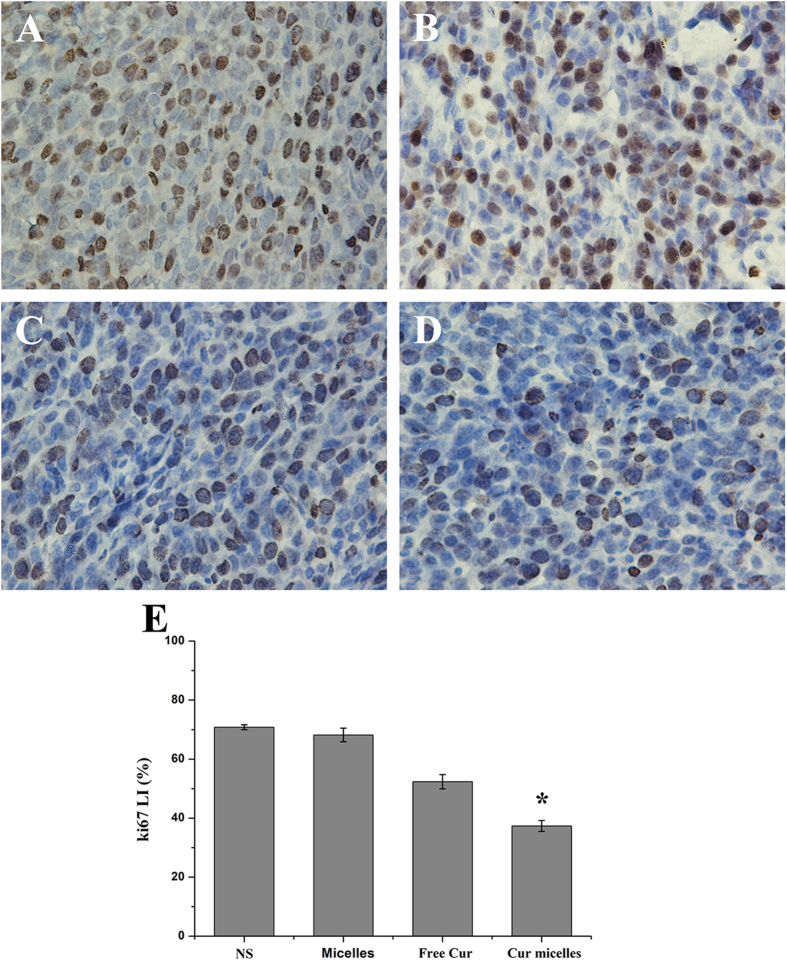
Ki-67 immunohistochemical staining of tumors. Representative Ki-67 immunohistochemical images of tumors: NS (**A**), blank micelles (**B**), free Cur (**C**), Cur micelles (**D**), Mean Ki-67 LI in each group (**E**).

We examined the proliferation activity of Cur micelles by immunohitochemical staining of Ki-67. According to Fig. 6A to D, staining for the cell proliferation marker Ki-67 showed markedly lower proliferation in subcutaneous CT26 model treated with Cur micelles compared with other groups. Additionally, the Ki-67 labeling index (LI) in Cur micelles (37.33 ± 1.85%) was significantly lower than free Cur (52.32 ± 2.41%, *p* < 0.05), blank micelles (68.15 ± 2.32%, *p* < 0.05), or NS group (70.82 ± 0.83%, *p* < 0.05), respectively (Fig. 6E).

### Quantitative Assessment of Apoptosis

**Figure 7:**
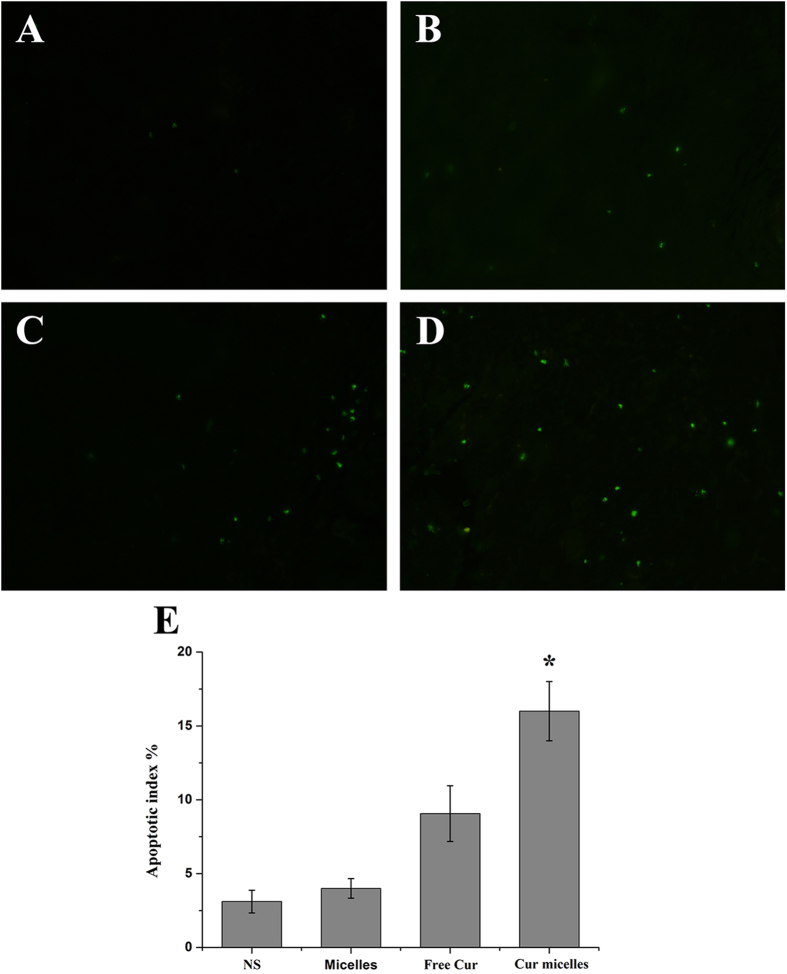
Apoptosis of colon cancer cells was examined using TUNEL analysis. Representative TUNEL immunofluorescent images of tumors: NS (**A**), blank micelles (**B**), free Cur (**C**), Cur micelles (**D**), Mean apoptotic index in each group (**E**).

To analyze the effect of Cur micelles on apoptosis in subcutaneous CT26 tumor, we measured by immunofluorescent TUNEL staining assays. In Fig. 7A to D, many strongly positive nuclei regarded as apoptotic could be observed in tumor section treated with Cur micelles, while such nuclei were rare in other groups. What’s more, Fig. 7E showed that the apoptosis index in Cur micelles group (16.05 ± 2.01%) was markedly higher than free Cur (9.07 ± 1.89%, *p* < 0.05), blank micelles (4.05 ± 0.66%, *p* < 0.05), or NS group (3.11 ± 0.76%, *p* < 0.05), respectively.

### Anti-angiogenesis Evaluation *In Vivo*

**Figure 8:**
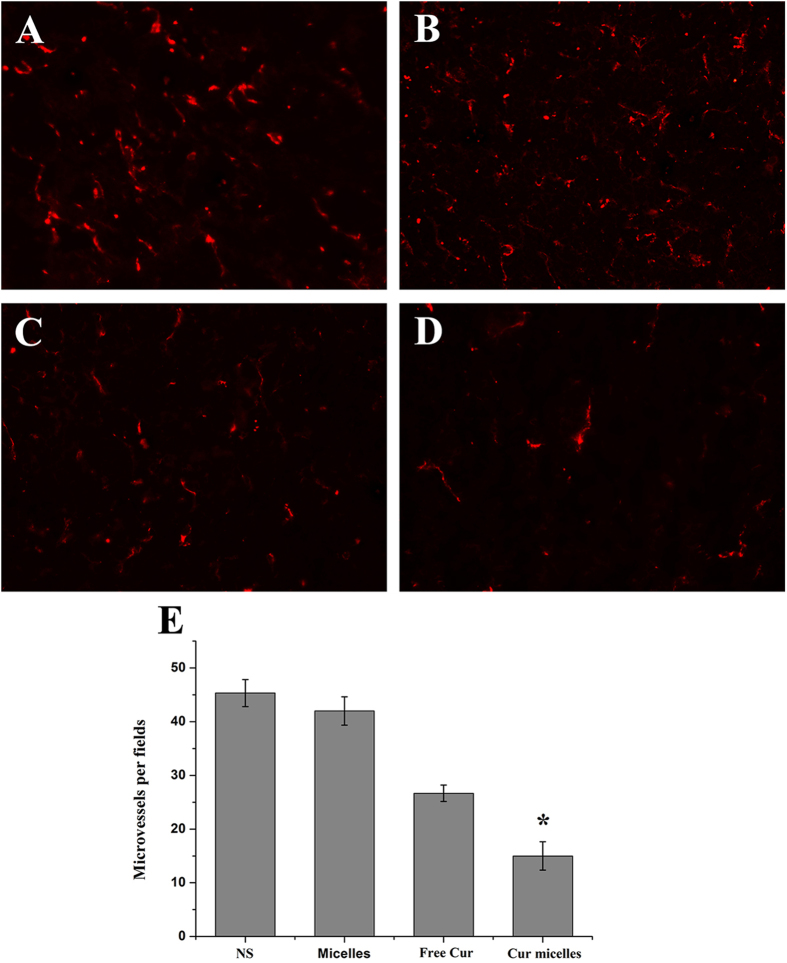
CD31 immunofluorescent staining of tumors. Representative CD31 immunofluorescent staining of tumors: NS (**A**), blank micelles (**B**), free Cur (**C**), Cur micelles (**D**), MVD in each group (**E**).

CD31 staining was performed on tumor tissue to estimate the microvessel density (MVD) as a measurement of tumor angiogenesis. As shown as in Fig. 8A to D, significant fewer immunoreactive microvessels were observed in tumor tissue treated with Cur micelles compared with other groups. Furthermore, MVD of tumor section was significantly lower in Cur micelles treatment (15.05 ± 2.65) than free Cur (26.67 ± 1.52, *p* < 0.05), blank micelles (42.05 ± 2.64, *p* < 0.05), or NS group (45.33 ± 2.51, *p* < 0.05), respectively (Fig. 8E)

### Toxicity Observation

During the whole observation period, no death and no gross side effects were investigated in each group after intravenous administration. We analyzed body weight variations in experimental period. In Supplementary Fig. 3A indicated that the mean weight of mice body had no significant difference among the four groups. However, compared with other groups the mice treated with free Cur were in a weak state and loss of weight.

To observe the side effects of each group, we did complete blood count (CBC) and serum chemistry profile test. According to the Supplementary Fig. 3B–H, no significant difference was observed among the four groups. All results indicated that the blood system, hepatic function, and renal function were not affected by administration of Cur micelles and free Cur. In addition, the histological examination of heart, lung, liver, spleen, kidney from Cur micelles, free Cur, blank micelles, and NS group were normal (Supplementary Fig. 4).

## Discussion

Cur, a yellow pigment derived from turmeric, has a wide range of anticancer properties^23^,^24^. However, the clinical application of Cur for some cancers has been restricted because of its extremely poor solubility, instability, and poor oral bioavailability as well as rapid metabolism and elimination from the body^17^,^18^,^25^. Different significant and promising nanocarriers for sustained and efficient Cur delivery, including micelles, conjugates, nanoparticles, liposomes and solid dispersions have focused on by many relevant researches^17^,^18^. Compared with other nanocarriers, micelles are much easier to prepare and also provide a flexible structure to develop multifunctional delivery systems^18^,^26^.

Micelles composed of amphiphilic block copolymers are widely used in the delivery of water insoluble agents with a size ranging from 20 to 100 nm in aqueous solution^27^,^28^. The hydrophobic segment of the amphiphilic molecules forms the core, while the hydrophilic segmnet forms the exterior shell^29^. Moreover, encapsulation of hydrophobic compounds in micelles have significant advantages such as improving water solubility, stability and distribution^29^,^30^. So far the amphiphilic block copolymers-based micelles have been extensively used for delivery of Cur, including amphiphilic methoxy poly (ethylene glycol)-b-poly (ε-caprolactone-co-p-dioxanone), mixed Pluronics P123 and F68, poly (ethylene oxide)-b-poly (ε-caprolactone) (PEO-PCL), and MPEG-PCL^31^,^32^,^33^,^34^. Researches from Ma *et al.* have developed amphiphilic block copolymer micelles for PEO-PCL as vehicles for the solubilization, stabilization, and controlled delivery of Cur^32^. PEO-PCL micelles encapsulated Cur were prepared by a co-solvent evaporation technique, which retained its cytotoxicity in B16-F10, a mouse melanoma cell line, SP-53, Mino and JeKo-1 human mantle cell lymphoma cell lines. In addition, the data suggested that the characteristics of micelles should depend on the polymerization degrees of PCL. However, the *in vivo* assay was not included. In this regard, other interesting studies have reported on the loading Cur in MPEG-PCL micelles by a one-step solid dispersion method without using any surfactants or toxic organic solvent^22^,^31^,^35^. Their findings revealed that Cur/MPEG-PCL micelles efficiently inhibited the angiogenesis on transgenic zebrafish model. Moreover, MPEG-PCL micelles-encapsulated Cur inhibited the growth of subcutaneous CT26 colon carcinoma model, 4T1 breast tumor model, and LL/2 pulmonary tumor model *in vivo*, which induced a stronger anticancer effect than free Cur. However, the major impediment towards clinical implication of MPEG-PCL micelles is the stronger crystallization of PCL resulting in the micelles instability. Some researches indicated that non-crystallization TMC in copolymer might prevent from forming the crystallization of PCL^21^,^36^.

Our previous work successfully synthesized the MPEG-P(CL-*co*-TMC) copolymers. The *in vitro* and *in vivo* safety evaluation demonstrated that the MPEG-P(CL-*co*-TMC) micelles could be a safe candidate for application in hydrophobic drug dilivery systems (DDSs)^37^. The MPEG-P(CL-*co*-TMC) micelles form a spherical core-shell architecture where MPEG of amphiphilic molecules forms the exterior shell, while the hydrophobic segmnt (the core) is random copolymer between the two hydrophobic monomers, TMC and ε-CL. Park *et al.* reported that the non-crystallization TMC in copolymers could protect the crystallization of PCL from the copolymers of ε-CL with TMC to improve the sol stability^21^. In XRD patterns, the characteristic crystalline peaks of the PCL (2*θ = *21.5 and 23.5) decreased in blank MPEG-P(CL-*co*-TMC) copolymer compared with blank MPEG-PCL copolymer, indicating that TMC in copolymer could inhibit the crystallization of PCL to improve the micelles stability. In a word, the major advantages of MPEG-P(CL-*co*-TMC) micelles are that they could form stable micelles by self-assembly method in aqueous medium and enhance the solubility and stability of poorly water-soluble drug^36^,^38^. Herein, we used MPEG-P(CL-*co*-TMC) micelles in this study to deliver Cur enhancing stability and solubility and improving anticancer effects *in vitro* and *in vivo*.

We loaded Cur into micelles of amphiphilic MPEG-P(CL-*co*-TMC) comploymers by a one-step solid dispersion method, which was much easier to prepare and scale up. Now, in this work, 15/85 as the Cur/MPEG-P(CL-*co*-TMC) copolymer ratio in feed was chosen for future application and characterized in detail. In brief, the theoretical amount of about 15 mg Cur and 85 mg MPEG-P(CL-*co*-TMC) copolymer were formed Cur micelles. The obtained Cur micelles had a small particle size of 27.6 ± 0.7 nm with PDI of 0.11 ± 0.05. According to the Eqs. (1) of DL and Eqs. (2) of EE, the results indicated that DL was 14.07 ± 30.94% and EE was 96.08 ± 3.23% measured by HPLC. The calculated EE was depended on the theoretical amount of 15 mg Cur added. During the observation period, the Cur included two parts: one part of Cur was encapsulated in polymeric micelles indicating a homogeneously transparent solution and the other part of Cur was non-encapsulated in micelles indicating the presence of precipitation. The precipitation of Cur could be filtrated through a 0.22 μm syringe filter. According to the results of stability on Cur micelles, the particle size distribution changes at 4 °C or 37 °C showed that the particle size of Cur-encapsulated micelles was relatively stable in 48 h. Moreover, DL and EE changes could a better display the stability on this delivery included the Cur-encapsulated and non-encapsulated Cur falling in line with the visible changes. In our previous work, the prepared Cur micelles kept at 4 °C were more stable within 48 h than Cur/MPG-PCL in feed ratio of 15/85 reported by Gong *et al.*^31^. The data of XRD indicated that Cur could form amorphous complex or disorded-crystalline phase in Cur micelles. The *in vitro* release study suggested that Cur micelles could release Cur slowly compared with free Cur. Therefore, Cur micelles could form an intravenous injectable aqueous formulation of Cur, circulate for a longer time *in vivo*, and accumulate in tumor tissue by the EPR effect.

To examine *in vitro* anticancer activity of Cur micelles, we performed on CT26 colon carcinoma cells using cytotoxicity study, apoptosis induction and cellular uptake of Cur micelles. The cytotoxicity study indicated that both free Cur and Cur micelles efficiently suppressed growth of CT26 colon carcinoma cells *in vitro* at 24 h and 48 h. Meanwhile, the results also suggested that blank MPEG-P(CL-*co*-TMC) micelles had little cytotoxic as a drug delivery carrier on CT26 cell line. Besides, Cur micelles induced a larger amount of apopototic cells than free Cur in study of fluorescence microscopy and FCM analysis *in vitro.* In this context, Cur micelles could enhance cellular uptake of Cur on CT26 cells to exert cytotoxicity and apoptosis effect. The hypothesis was confirmed by the experimental results of cellular uptake. Thus, our findings demonstrated that the fluorescence intensity of CT26 cells treated with Cur micelles was much stronger than free Cur after 2 h and 4 h incubation using fluorescence microscopy and FCM.

We prepared injectable Cur micelles which are able to be administered systemically in subcutaneous CT26 colon carcinoma model. The *in vivo* study confirmed that Cur micelles induced a stronger anticancer effect than free Cur. The mechanisms of Cur micelles inhibited growth of colon cancer could include suppression of tumor cell proliferation, induction tumor cell apoptosis and inhibition of tumor angiogenesis. Furthermore, Cur micelles at its therapeutic dosage have a few toxicity to normal tissue, including heart, lung, liver, kidney, spleen, and bone narrow. In contrast, compared with other groups the mice treated with free Cur were in a weak state and loss of weight. In spite of the safety of Cur is well-known in anticancer agents, free Cur dissolved in organic solvents for intravenously injected in mice might have side effects. Besides, Cur micelles as nano-sized particles can easily enhance drug accumulation in tumor site by the EPR effect, while decrease drug extravasation from normal vessels into normal tissues compared with free Cur^20^,^39^. In summary, Cur loaded in MPEG-P(CL-*co*-TMC) micelles had a promising application in treating colon carcinoma.

## Conclusions

Encapsulation of Cur in MPEG-P(CL-*co*-TMC) micelles were prepared and assigned for therapy on CT26 colon carcinoma *in vitro* and *in vivo*. The obtained Cur micelles inhibited the growth of CT26 cells *in vitro* through increasing apoptosis induction and cellular uptake. In addition, Cur micelles were much more effective than free Cur in inhibiting growth of CT26 colon tumor. Few side effects were found in Cur micelles. Therefore, Cur micelles are a promising aqueous formulation of Cur for intravenous application with enhanced antitumor activity, which may be a potential treatment strategy for colon cancer in the future.

## Methods

### Materials, Cell lines, Animals

Curcumin (Sigma, USA, the purity more than 98%), monomethyl poly (ethylene glycol) (MPEG, *M*_n_ = 2000, Aldrich, USA), 1,3-trimethylene carbonate (TMC, Huizhou Foryou Medical Devices, China), ε-caprolactone (ε-CL, Alfa Aesar, USA), stannous octoate (Sn(Oct)_2_, Aldrich), methanol and acetic acid (HPLC grade, Fisher Scientific, UK), methyl thiazolyl tetrazolium (MTT, sigma, USA), propidium iodide (PI, sigma, USA), and 4’,6-diamidino-2-phenylindole 2hci (DAPI, sigma, USA) were used without further purification.

The CT26 colon carcinoma cells were obtained from the American Type Culture Collection (ATCC, Rockville, MD), which cultured in RPMI 1640 (Gibco, USA) supplement with 10% fetal bovine serum (FBS, Caoyuan lvye, Huhht, China). All cells were incubated at 37 °C with a humidified 5% CO_2_ atmosphere.

Female BALB/c mice, weighing 22 ± 2 g, were used for *in vivo* anti-tumor tests. The animals were purchased from Vital River Laboratories (Beijing, China) and quarantined for a week before treatment. Mice were supplied with pathogen-free conditions, and fed with standard laboratory chow and water *ad libitum*.

### Ethics Statement

All animal work were conducted under the approved guidelines of Sichuan University (Chengdu, China) and approved by the Animal Care Committee of Sichuan University (Chengdu, China).

### Synthesis of the MPEG-P(CL-*co*-TMC) Copolymer

MPEG-P(CL-*co*-TMC) copolymer with MPEG-PCL-TMC ratio (2000-1700-500) was synthesized by ring-opening polymerization of ε-CL and TMC initiated by MPEG^37^. Briefly, calculated the quality of TMC (2.5 g), MPEG (10 g), and ε-CL (8.5 g) were introduced into vessel under dry nitrogen atmosphere. The reaction was catalyzed by stannous octoate and allowed to keep at 130 °C for 24 h. The obtained MPEG-P(CL-*co*-TMC) copolymer was determined by FTIR (NICOLET 200SXV, Nicolet, USA), ^1^*H*-NMR (Varian 400 spectrometer, Varian, USA), and gel permeation chromatography (GPC, Agilent 110 HPLC, Agilent, USA).

### Preparation and Characterization of Cur Micelles

Cur micelles were obtained through a single-step solid dispersion method^22^. In brief, 85 mg MPEG-P(CL-*co*-TMC) copolymer and 15 mg Cur were co-dissolved in 5 mL of acetone under mild stirring and then co-evaporated in a rotary evaporator at 60 °C. Subsequently, the coevaporation was dissolved in NS at 60 °C to form Cur micelles. The excess drug was removed by filtration through a 0.22 μm syringe filter (Millex-LG, Millipore Co., USA), and the obtained Cur micelles solution was lyophilized and kept at 4 °C before use.

The amount of Cur entrapped in loaded micelles was estimated by a high performance liquid chromatography (HPLC, Waters Alliance 2695). Detection was taken on a Waters 2996 detector. For this, 20 μL of the sample was injected automatically in the injection port and analyzed in the mobile phase consisting of methanol/0.3% acetic acid (80/20, v/v). The flow rate was set at 1 mL/min with C_18_ column (4.6 × 150 mm-5 μm, Sunfire Analysis column). The detection wave-length for Cur was set at 420 nm, and the elution time of Cur was 5.272 min under chromatographic conditions described above.

Drug loading (DL) and encapsulation efficiency (EE) of the Cur micelles were estimated as follows. In detail, 10 mg of lyophilized Cur micelles were dissolved in 0.1 mL of acetonitrile. The concentration of Cur micelles in solution was determined by HPLC and calculated using the following Eqs. (1) and (2):


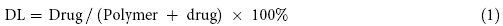



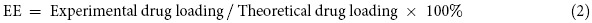


Malvern Nano-ZS 90 laser particle size analyzer was used to measure the particle size distribution and zeta potential of Cur micelles. All reported experimental results were performed in triplicate as a mean size ± standard deviation (SD).

The stability of Cur micelles aqueous solution kept at 4 °C and 37 °C for 48 h was evaluated qualitatively by the observation of aggregates. The presence of precipitation implied instability of the Cur micelles. In addition, the stability on this delivery system was monitored for the particle size distribution, DL and EE changes. At particular time intervals, 50 μL of simple was colletcted and filtrated through a 0.22 μm syringe filter, and added 950 μL of methanol for quantitative analysis of Cur. Then, the particle size distribution was measured by Malvern Nano-ZS 90 laser particle size analyzer. DL and EE of the Cur micelles were determined by HPLC and calculated using the Eqs. (1) and (2).

XRD patterns of free Cur, blank MPEG-P(CL-*co*-TMC) copolymer, Cur + MPEG-P(CL-*co*-TMC) copolymer, and Cur micelles were detected by a diffractometer (X’Pert Pro, Philips, Netherlands) equipped with a rotating target X-ray tube and a wide-angle goniometer.

We investigated the morphological characteristics of the prepared Cur micelles using TEM.

### *In Vitro* Release Study

The *in vitro* release studies of Cur micelles were performed by a dialysis method. A volume of 0.5 mL of Cur micelles or free Cur (1 mg/mL) was placed into dialysis bags with 3.5 kDa molecular weight cutoff. The dialysis bags were immersed into 10 mL of PBS (pH 7.4) containing 0.5% v/v tween-80 and kept in a shaker at 37 °C at 100 rpm. At particular time intervals, 1 mL of sample was collected and stored at −20 °C until analysis. Then the remaining release media were removed and replaced by 10 mL of the pre-warmed fresh buffer. Subsequently, the amount of Cur in the incubation medium was quantified by HPLC as described previously. The experimental results were repeated three times, and all data were expressed as mean value ± SD.

### Cytotoxicity Evaluation of Cur Micelles

*In vitro* cytotoxicity evaluation of free Cur, Cur micelles, and blank micelles for CT26 cell line was determined by the MTT assay. Free Cur dissolved in dimethyl sulfoxide (DMSO). Especially, the final concentration of DMSO in free Cur on CT26 cells was less than 0.1%. CT26 cells were seeded at a density of 2 × 10^3^ cells in 96-well plates and incubated overnight. Subsequently, the cells were exposed to a series of free Cur, Cur micelles, or blank micelles at different concentrations. Cells were incubated with treatment for 24 h and 48 h. At the end, the percentage of viable cells relative to untreated control was determined on the basis of the mitochondrial conversion of 3-(4,5-dimethylthiazol-2-yl)-2,5-diphenyltetrazolium bromide to formazan. The results were expressed in 5 measurements as mean value ± SD.

### Apoptosis Induction of Cur Micelles

The induction of apoptosis was studied by fluorescence microscopy and FCM analysis. DAPI staining was used to identify apoptosis by fluorescence microscopy. CT26 cells were cultured in 6 well plates with acid etched glass coverslips containing 2 mL of grown medium in triplicate overnight at 37 °C. After treatment with free Cur (7.5 μg/mL), Cur micelles (7.5 μg/mL) and blank micelles for 48 h, the grown medium was removed and the treated cells were carefully washed twice with PBS. Medium without Cur was added as control. Subsequently, samples were fixed with cold acetone, washed again with PBS, stained with DAPI, and examined using a fluorescence microscopy (DM2500, LEICA, Germany).

PI were performed to stain the CT26 cells and the percentage of cell apoptosis was determined by FCM. Cells were seeded in 6-well plates overnight. Then, the cells were exposed to free Cur (7.5 μg/mL), Cur micelles (7.5 μg/mL) and blank micelles for 48 h. Meanwhile, cells cultured in medium without treatment reagents were used as control. The treated cells were harvested, washed with PBS, fixed with 70% ethanol for 30 min, and stained with PI. Finally, the stained samples were analyzed by a flow cytometer (FACSCalibur, BD, USA), at least 10 000 cells were harvested from each sample. The PI fluorescence from individual cells was detected, while excitation was with the 488 nm line of an argon laser and emission fluorescence measured was larger than 630 nm. The percentage of apoptotic was calculated as number of sub-G1 (apopototic) cells/total number of cells in there measurements by the software of BD FACSCalibur.

### Cellular Uptake of Cur Micelles

Cellular uptake of Cur micelles was also measured by fluorescence microscopy and FCM analysis. CT26 cells were plated onto acid etched glass coverslips at a density of 2 × 10^5^ cells/mL in 6-well plates and cultured in 2 mL of medium overnight. The attached cells were treated with an equivalent dose (7.5 μg/mL) of free Cur and Cur micelles in serum-free medium. Meanwhile, cells were used as control treated with only serum-free medium. After incubation at 37 °C for 2 h and 4 h, the cells were washed twice with PBS, fixed with cold acetone, washed again with PBS, stained with DAPI, and imaged using a fluorescence microscopy. In FCM analysis, the cells were treated as described above and analyzed by FCM. The excitation was with the 488 nm line of an argon laser and the emission fluorescence of Cur-derived between 515 nm and 545 nm was detected.

### *In Vivo* Antitumor Activity

*In vivo* antitumor activity of Cur micelles was investigated by subcutaneous CT26 colon tumor model. BALB/c female mice were subcutaneously injected with 100 μL of cell suspension containing 5 × 10^5^ CT26 in the right flank at day 0. When tumors were detectable on day 6, the mice were randomly assigned in four groups (6 mice per group). The four groups were intravenously injected with NS (control), blank micelles (336 mg/kg), free Cur (50 mg/kg Cur equivalent) and Cur micelles (50 mg/kg Cur equivalent) daily for there weeks, respectively. Free Cur was dissolved in the organic solvent consisting of ethanol/ tween-80/normal saline (7.5/7.5/85, v/v/v) achieving the final concentration for *in vivo* injection. Tumor size (*ab*^2^/2; a and b are the long and short axes of the tumor) and body weight were measured every three days during the whole observation period. On day 27, the control mice began to die. Visceral organs (heart, lung, liver, spleen, and kidney) and tumor tissues in each group were harvested and either preserved in 4% paraformaldehyde or snap frozen at −80 °C.

### Immunohistochemical Determination of Ki-67

To assess the tumor cell proliferation, we investigated Ki-67 staining by the labeled streptavidin-biotin method. The primary antibody was rat anti-mouse monoclonal antibody Ki-67 (CST, USA) and the secondary antibody was biotinylated goat anti-rat immunoglobulin (Abcam, USA). In the Ki-67 staining tests, all the five tumors in each group were sectioned. In tumor tissue sections, five equal-sized fields were randomly chosen and analyzed. The Ki-67 labeling index was calculated as number of Ki-67 positive cells/total number of cells counted in five randomly selected areas in each tumor sample by two independent investigators in a blinded fashion.

### Quantitative Determination of Apoptosis

Following the protocol of manufacturer, terminal deoxynucleotidyl transferase-mediated nick-end labeling (TUNEL) staining was conducted by an *in situ* cell death detection kit (DeadEnd^TM^ Fluorometric TUNEL System, Promega, Madison, USA) to test the apoptosis cells in tumor^40^. TUNEL staining is based on the enzymatic addition of digoxigenin-nucleotide to the nicked DNA by recombinant terminal deoxynucleotidyl transferase (rTdT). In the TUNEL staining tests, all the five tumors in each group were sectioned. In tumor tissue sections, five equal-sized fields were randomly chosen and analyzed. The apoptotic index was calculated as number of the TUNEL-positive cells/total number of cells in five randomly selected tumor areas in each tumor sample by two independent investigators in a blinded fashion.

### Detection of Microvessel Density (MVD)

Immunofluorescent analysis of neovascularization maker CD31 in tumor tissue determined the antiangiogenesis activity of Cur. Following the protocol, frozen sections 4–5 μm thick of tumor tissue were fixed in cold acetone, washed with PBS, stained with rabbit anti-mouse CD31 polyclonal antibody (Abcam, USA) overnight at −4 °C, washed twice with PBS, and incubated with a FITC-conjugated second antibody (Abcam, USA) for 40 min. In the CD31 staining tests, all the five tumors in each group were sectioned. In tumor tissue sections, five equal-sized fields were randomly chosen and analyzed. The detection of microvessel density was evaluated by calculating the average number of small CD31-positive microvessels in five randomly selected tumor areas in each tumor sample by two independent investigators in a blinded fashion.

### Toxicity assessment

To evaluate the possible toxicity and side effects, all mice were continuously observed after intravenous administration, including the general conditions (the activity, skin, fair, feces, secretion, behavior, energy, and other clinic signs), body weight, and mortality^41^,^42^. Before sacrificed, blood was obtained, and used for CBC and serum chemistry profile test. The main organs (heart, lung, liver, spleen, and kidney) were harvested, fixed in 4% paraformaldehyde, and stained with H&E for histopathological examination.

### Statistical analysis

Statistical analyses were performed using one-way analysis of variance (ANOVA). All results were expressed as mean ± SD, and the differences were considered significant for *p* values of less than 0.05. The statistical analyses were carried out using SPSS 17.0 software (Chicago, IL, USA).

## Author Contributions

C.G. and C.Y. designed the experiments, and the research funds were supported by C.G.; X.Y. carried out experiments, analyzed the data, and wrote the manuscript; Q.W. and C.G. corrected the manuscript; L.S., Z.L., and Z.W. participated in the animal experiments; N.W. and L.L. participated in the immunohitochemical and immunofluorescent assays; L.S. and T.H. participated in synthesizing the polymer; N.L. and X.L. participated in analyzing the data. All authors approved and read the final manuscript.

## Additional Information

**How to cite this article**: Yang, X. *et al.* Curcumin-Encapsulated Polymeric Micelles Suppress the Development of Colon Cancer *In Vitro* and *In Vivo*. *Sci. Rep.* **5**, 10322; doi: 10.1038/srep10322 (2015).

## Supplementary Material

Supplementary Information
