# Supplementary material for: Curcumin-Encapsulated Polymeric Micelles Suppress the Development of Colon Cancer In Vitro and In Vivo
Source: Sci Rep. 2015 May 18;5:10322. doi: 10.1038/srep10322 (PMC4434844; doi:10.1038/srep10322)
Supplement: Supplementary Information [file srep10322-s1.doc]

Supplementary information

Curcumin-Encapsulated Polymeric Micelles Suppress the Development of Colon Cancer *In Vitro* and *In Vivo*

Xi Yang^1,+^, Zhaojun Li^1,+^, Ning Wang^1^, Ling Li^1^, Linjiang Song^1^, Tao He^1^, Lu Sun^1^, Zhihan Wang^1^, Qinjie Wu^1^, Na Luo^2^, Cheng Yi^1^,*, Changyang Gong^1^,*

^1^ Department of Medical Oncology, Cancer Center, State Key Laboratory of Biotherapy/Collaborative Innovation Center of Biotherapy, West China Hospital, Sichuan University, Chengdu, 610041, P. R. China

^2^ School of Medicine, Nankai University, Tianjin, 300071, China

^+^ These authors contributed equally.

* To whom correspondence should be addressed (C Gong and C Yi). E-mail: chygong14@163.com and yicheng6834@163.com

Supplementary Figure 1


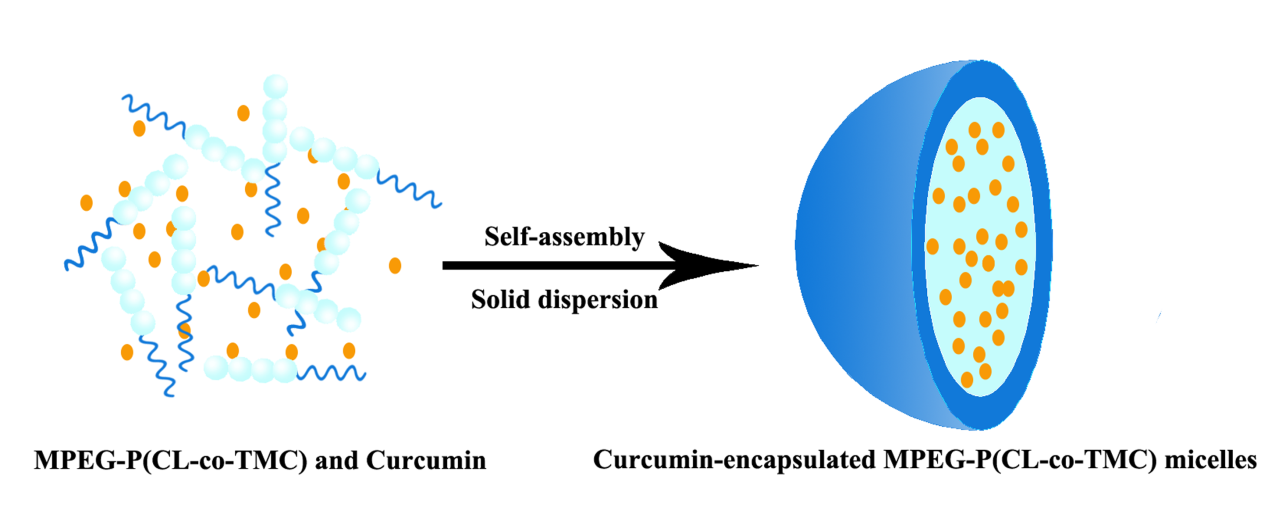


**Figure 1** Preparation scheme of Curcumin-encapsulated MPEG-P(CL-*co*-TMC) micelles using a one-step solid dispersion method.

Supplementary Figure 2


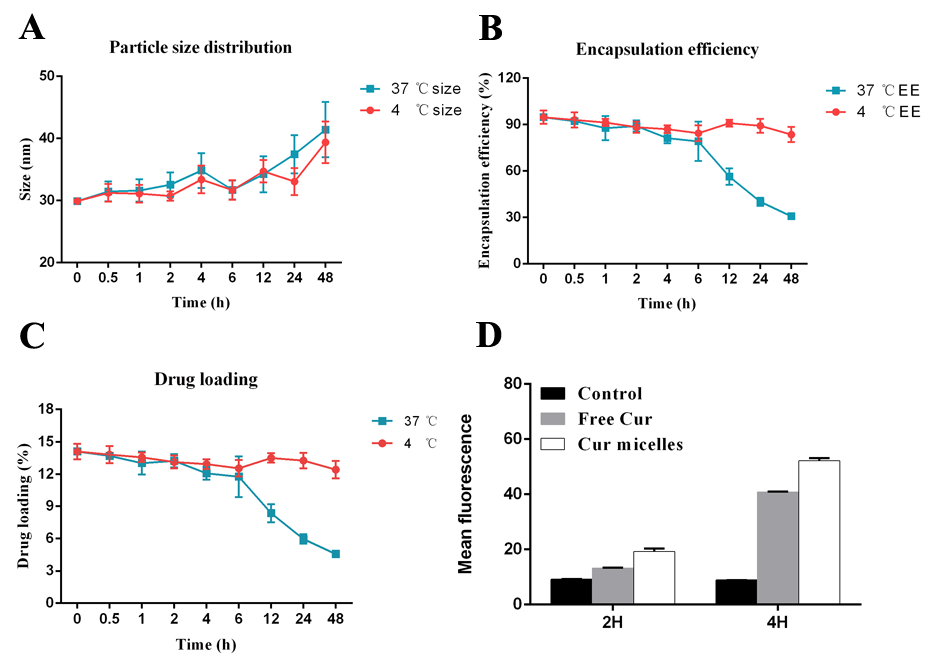


**Figure 2** The stability of Cur micelles on this delivery system was monitored for the particle size distribution changes (A), drug loading (B) and encapsulation efficiency (C) changes. The mean fluorescence intensity detected by the FCM in each group (D).

Supplementary Figure 3


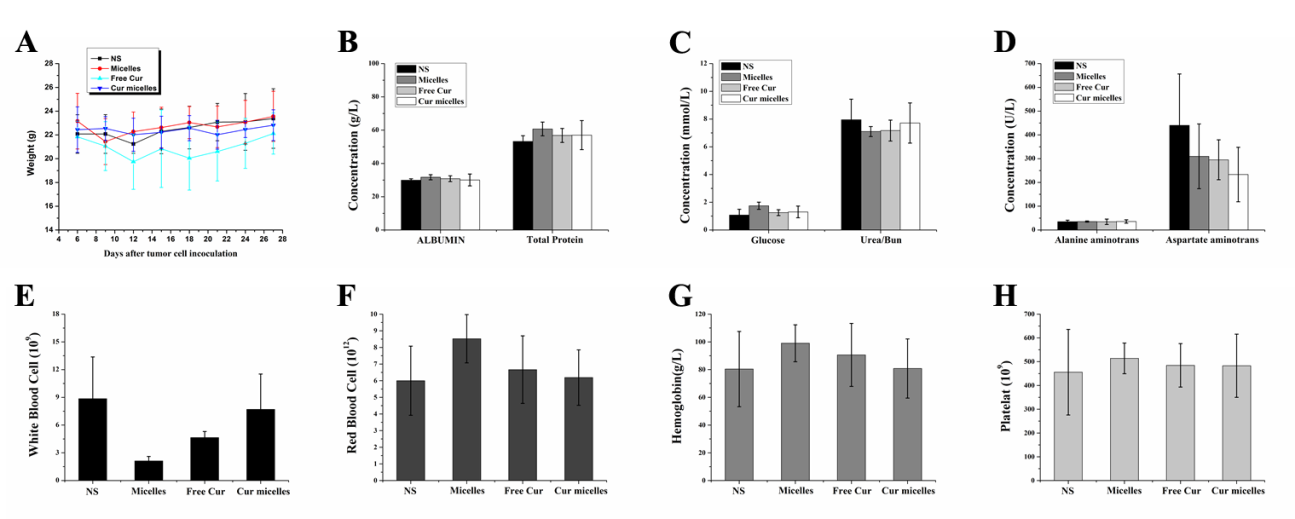


**Figure 3** Toxicity side effects were observed in each group after intravenous administration. Mice body weight of each group in observation period (A). Serum chemistry profile and complete blood count in subcutaneous CT26 model after intravenous administration (B-H). B-H: B, albumin and total protein; C, glucose and urea/bun; D, alanine aminotrans (ALT) and aspartate aminotrans (AST); E, white blood cell; F, red blood cell; G, hemoglobin; H, platelet.

Supplementary Figure 4


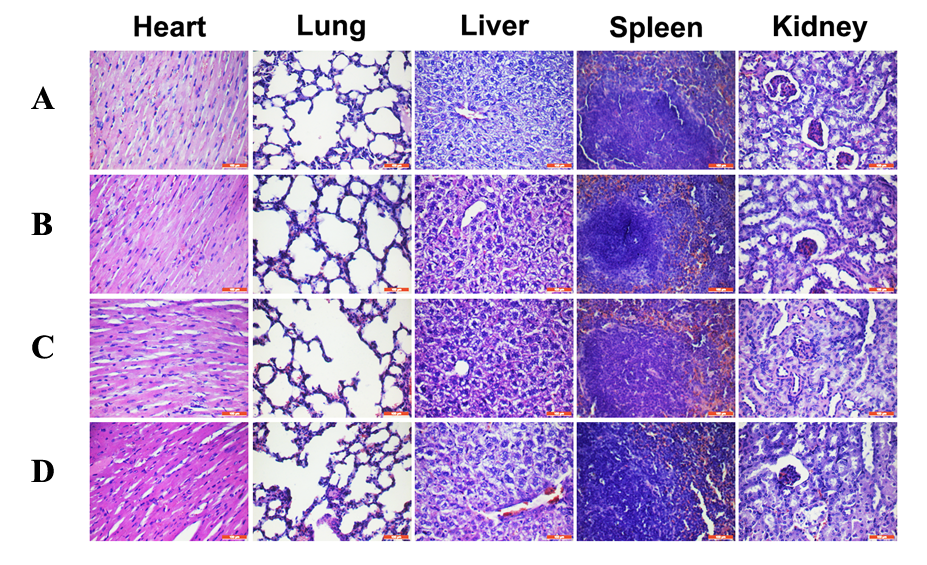


**Figure 4** H&E staining of major organs (heart, lung, liver, spleen, and kidney) after intravenous administration: NS (A), blank micelles (B), free Cur (C), Cur micelles (D).
